# Combination of Inhibitors of USP7 and PLK1 has a Strong Synergism against Paclitaxel Resistance

**DOI:** 10.3390/ijms21228629

**Published:** 2020-11-16

**Authors:** Sol-Bi Shin, Chang-Hyeon Kim, Hay-Ran Jang, Hyungshin Yim

**Affiliations:** Department of Pharmacy, College of Pharmacy, Institute of Pharmaceutical Science and Technology, Hanyang University, Ansan, Gyeonggi-do 15588, Korea; solbi@hanyang.ac.kr (S.-B.S.); shinkher@hanyang.ac.kr (C.-H.K.); ggoma337@hanyang.ac.kr (H.-R.J.)

**Keywords:** USP7, PLK1, taxane, chemoresistance, combination

## Abstract

USP7 is a promising target for the development of cancer treatments because of its high expression and the critical functions of its substrates in carcinogenesis of several different carcinomas. Here, we demonstrated the effectiveness of targeting USP7 in advanced malignant cells showing high levels of USP7, especially in taxane-resistant cancer. USP7 knockdown effectively induced cell death in several cancer cells of lung, prostate, and cervix. Depletion of USP7 induced multiple spindle pole formation in mitosis, and, consequently, resulted in mitotic catastrophe. When USP7 was blocked in the paclitaxel-resistant lung cancer NCI-H460^TXR^ cells, which has resistance to mitotic catastrophe, NCI-H460^TXR^ cells underwent apoptosis effectively. Furthermore, combination treatment with the mitotic kinase PLK1 inhibitor volasertib and the USP7 inhibitor P22077 showed a strong synergism through down-regulation of *MDR1/ABCB1* in paclitaxel-resistant lung cancer. Therefore, we suggest USP7 is a promising target for cancer therapy, and combination therapy with inhibitors of PLK1 and USP7 may be valuable for treating paclitaxel-resistant cancers, because of their strong synergism.

## 1. Introduction

Approximately 80% of intracellular proteins are bound to ubiquitin, a labeling protein that signals for degradation by the proteasome complex that recognizes it. Ubiquitin-specific-processing protease 7 (USP7), one of the deubiquitinating enzymes, functions in the ubiquitin-proteasome machinery. It regulates the stability of various proteins, thereby affecting their physiological functions in cells and playing a wide role in signal transduction processes in not only the cell cycle, but also in the stress response, DNA repair, and apoptosis, depending on the cellular substrates and context [1,2,3,4,5,6,7,8]. The multiple roles of USP7 have been studied in various carcinomas, including prostate [1], lung [9,10,11], breast [12], ovary [13], brain [14], and colon [15]. Its high expression is directly associated with carcinogenesis in prostate [1], lung [9], breast [12], glioma [14], and colon cancer [15], inducing proliferation of these cancer cells. In particular, it has been reported that overexpression of USP7 is closely related to the malignancy of prostate cancer [1]. Based on these oncopathological characteristics, selective inhibitors against the catalytic activity of USP7 have been actively developed, such as HBX-41108 [16], P22077 [17], and P5091 [18,19]. They are effective for suppressing the growth of neuroblastoma, colon cancer, and ovarian cancer [16,17,18,19,20]. Therefore, based on the current literature, pharmacological inhibitors of USP7 are promising anticancer agents in various carcinomas, depending on the cellular context.

Taxanes, such as paclitaxel and docetaxel, are microtubule stabilizing agents which induce mitotic catastrophe in cancer cells [21,22]. They are first-line chemotherapeutic agents for several carcinomas of lung [23], prostate [24], breast [25], and ovary [26,27]. Although the therapeutic efficacy of taxanes is high for cancer treatment, often chemoresistance can develop in some patients. Chemoresistance is a main limitation to chemotherapy, and one of the major causes of mortality during cancer treatment. Since taxanes are used frequently for the patients who have advanced prostate, breast, or lung carcinoma [23,24,25,28], the acquisition of taxane resistance demands rapidly finding other therapeutic targets to facilitate taxane-resistant cancer treatment.

Previously, we found that USP7 expressed in mitosis interacts with 53BP1 and PLK1 [2]. These observations lead us to hypothesize that targeting USP7 may be useful for cancer treatment, especially in taxane-resistant carcinoma. Based on this concept, we evaluated the expression and inhibitory effects of USP7 in several cancer cells of the prostate, lung, and cervix. Here, we found that USP7 inhibition induces mitotic catastrophe and apoptosis effectively in paclitaxel-resistant non-small cell lung cancer (NSCLC), alone or in combination with the mitotic kinase PLK1 inhibitor volasertib.

## 2. Results

### 2.1. Depletion of USP7 Induces Apoptosis in Several Carcinoma

**Figure 1:**
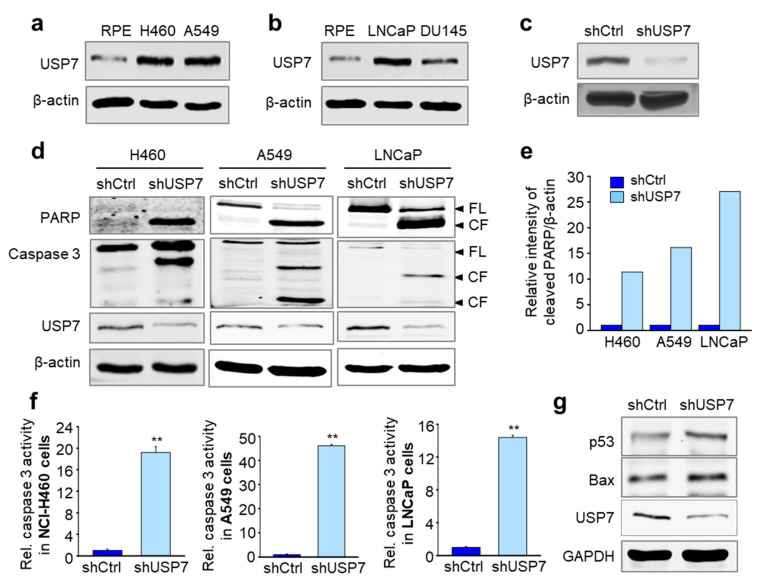
Depletion of USP7 induces apoptosis by activation of caspase 3 in several cancer cell lines. (**a**,**b**) Immunoblot analysis was performed to detect the levels of USP7. Cell lysates from non-transformed hTERT-RPE cells and lung cancer (**a**) or prostate cancer (**b**) cells were prepared to determine the levels of USP7 using a specific anti-USP7 antibody. (**c**) USP7 was depleted in LNCaP cells using USP7 shRNA. Immunoblot analysis was performed to detect the levels of USP7. (**d**) Immunoblot analysis was performed to evaluate levels of USP7, cleaved PARP, and cleaved caspase 3 in NCI-H460 (left), A549 (middle), and LNCaP (right) cells when USP7 was depleted. (**e**) The band intensity values from the immunoblots in Figure 1d were quantified using LI-COR Odyssey software (Li-COR Biosciences), normalized, and plotted. (**f**) Cell lysates from NCI-H460 (left panel), A549 (middle panel), and LNCaP (right panel) cells were subjected to a fluorometric caspase 3 activity assay. The means ± SDs (error bars) of data from at least three experiments are shown. (** *p* ≤ 0.01) (**g**) Immunoblot analysis was performed to evaluate the levels of p53 and Bax in USP7-depleted A549 cells.

To evaluate the expression of USP7 in both normal cells and lung and prostate cancer cells, we performed immunoblotting using samples isolated from hTERT-immortalized retinal pigment epithelial (hTERT-RPE) normal cells, NCI-H460 and A549 NSCLC cells, and LNCaP and DU145 prostate cancer cells (Figure 1a,b). As expected, the levels of USP7 were higher in lung and prostate cancer cells than in normal hTERT-RPE cells (Figure 1a,b). Next, we performed loss-of-function experiments to clarify whether USP7 induces cell death in lung and prostate cancer cell lines. For this, USP7 was depleted using lentiviral shRNA targeting human USP7 and the depletion was confirmed by immunoblot analysis (Figure 1c). Following USP7 depletion, we observed an increase in the proteolytic cleavage of the endogenous nuclear protein Poly (ADP-ribose) polymerase (PARP) and caspase 3, hallmarks of apoptosis, in NCI-H460 and A549 cells and LNCaP cells (Figure 1d,e). In addition, we measured the activity of caspase 3 in USP7-depleted cancer cells using a caspase 3-specific fluorogenic substrate (Figure 1f). Caspase 3 activities were markedly increased following USP7 depletion in NCI-H460, A549, and LNCaP cells (Figure 1f). To define apoptotic signaling induced by USP7 depletion, the levels of p53, apoptotic transcriptional activator, were determined by immunoblot analysis in USP7-depleted A549 cells (Figure 1g). The levels of p53 were higher in USP7-depleted cells than those of control, indicating that USP7 depletion up-regulates p53. In addition, the levels of Bax, one of downstream targets of p53 and pro-apoptotic factor [29], were higher in USP7-depleted cells than those of control, showing that USP7 depletion up-regulates p53 and its downstream pro-apoptotic factor Bax (Figure 1g). Therefore, loss of USP7 effectively induced apoptotic cell death in these lung and prostate cancer cells.

### 2.2. USP7 Was Highly Expressed Concomitantly with the Mitotic Factors in Tumor Tissues from Prostate and Non-Small Cell Lung Cancer

**Figure 2:**
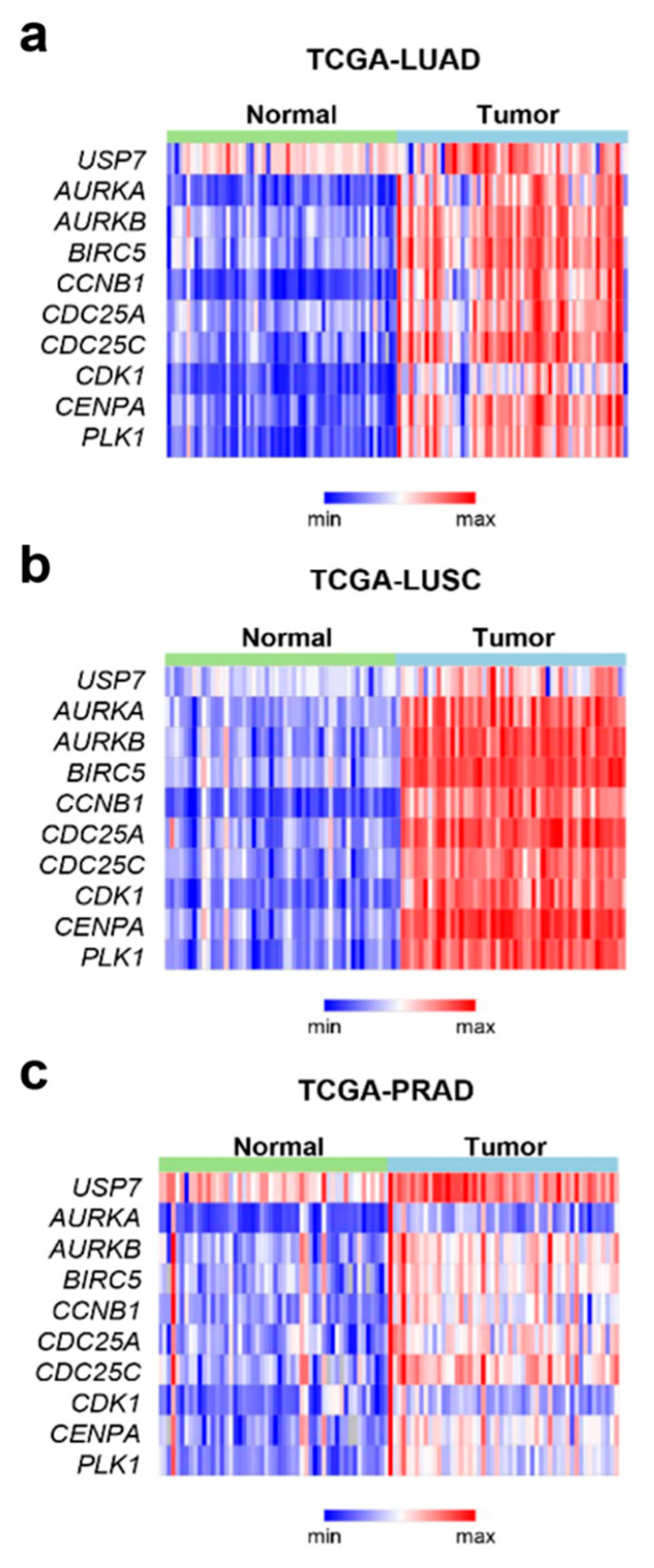
The gene expression profile of *USP7* in paired normal and tumor tissues from several types of carcinomas. Heat maps were generated using patient datasets from TCGA of lung adenocarcinoma (TCGA-LUAD) (**a**), lung squamous cell carcinoma (TCGA-LUSC) (**b**)**,** and prostate adenocarcinoma (TCGA-PRAD) (**c**). Heat maps show the expression profiles of mitotic genes, including *USP7*, *AURKA, AURKB, BIRC5, CCNB1*, *CDC25A, CDC25C, CDK1, CENPA,* and *PLK1*.

According to our previous study [2], inhibition of USP7 causes ubiquitination of 53BP1 and its destabilization, which induces multiple spindle pole formation in cells and subsequent mitotic defects. To understand whether the expression of USP7 is required for tumor progression in cancer patients and the association between the expression of mitotic factors and *USP7*, we analyzed the genomes of patients with lung adenocarcinoma (Figure 2a), lung squamous cell carcinoma (Figure 2b), and prostate adenocarcinoma (Figure 2c), using data from The Cancer Genome Atlas (TCGA). In brief, we found that the *USP7* was highly expressed concomitantly with the mitotic factors such as *PLK1, CCNB1, CDC25A,* and *AURKB* in prostate and lung cancer. The levels of *USP7* and mitotic markers, including *AURKB*, *CCNB1*, and *PLK1*, were higher in tumor samples than in normal tissues. Our analysis revealed that the expression patterns of *AURKB*, *CCNB1*, *PLK1*, and *USP7* were all higher in tumor tissues than in normal tissues, although their basal levels were different in each cancer (Figure 2).

### 2.3. Loss of USP7 Results in the Formation of Multiple Spindle Poles, Consequently Inducing Mitotic Catastrophe

**Figure 3:**
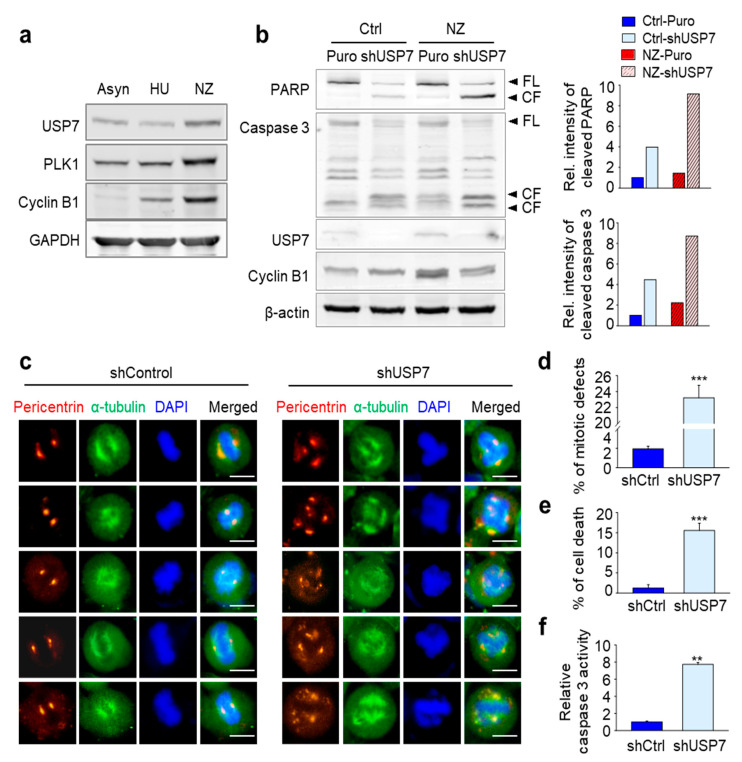
Loss of USP7 results in the formation of multiple spindle poles and, consequently, mitotic catastrophe. (**a**) HeLa cells were treated with 2 mM hydroxyurea (HU) or 100 ng/mL nocodazole (NZ) for 12 h. Asynchronized cells (Asyn) were prepared for comparison. Cell lysates were prepared for Western blot with anti-USP7, anti-PLK1, anti-cyclin B1, and anti-GAPDH antibodies. (**b**) HeLa cells were treated with 100 ng/mL nocodazole (NZ) for 12 h to synchronize mitosis, and then cells were infected with lentiviral USP7 shRNA for 48 h. Then, cell lysates were subjected to immunoblotting. The levels of cleaved PARP and cleaved caspase 3 were detected using anti-PARP and anti-caspase 3 antibodies (left panel). The relative band intensities were quantified using LI-COR Odyssey software (Li-COR Biosciences), normalized, and plotted (right panel). (**c**) HeLa cells grown on coverslips were infected with lentiviral USP7 shRNA or control virus. At 9 h after release from the double thymidine block, cells were fixed with 4% paraformaldehyde and stained with anti-pericentrin (Abcam; red) and anti-α-tubulin (Sigma; green). DAPI was used for staining nuclei. Bar scale, 10 µm (**d**) Quantification of mitotic-defective cells with multipolar spindles with greater than two pericentrin foci in USP7-depleted cells; *n* > 1000 cells. (**e**) Quantification of apoptotic cells with characteristic apoptotic nuclear morphology such as nuclear condensation in USP7-depleted cells; *n* > 1000 cells. (**f**) Cell lysates were subjected to a fluorometric caspase 3 activity assay. The means ± SDs (error bars) of data from at least three experiments are shown. (** *p* ≤ 0.01; *** *p* ≤ 0.001).

Next, we wanted to understand whether the apoptosis induced by USP7 depletion is caused by a mitotic catastrophe, similarly with the multiple spindle pole formation in cells and subsequent mitotic defects induced by destabilization of 53BP1. First, we evaluated changes in USP7 levels during the cell cycle. HeLa cells were treated with hydroxyurea and nocodazole for the synchronization of S phase and G2/M phase, respectively (Figure 3a). USP7 was up-regulated in the G2/M phase when compared to the S phase, in a manner similar to mitotic factors PLK1 and cyclin B1 (Figure 3a). Then, to observe whether depletion of USP7 induces apoptosis more effectively in the G2/M phase, when USP7 is up-regulated, mitotic cells synchronized with nocodazole were treated with USP7 shRNA. In Figure 3b, the levels of cleaved PARP and cleaved caspase 3 increased approximately 9-fold in USP7-depleted, nocodazole-treated cells compared with control cells (Figure 3b). Mitotic synchronized cells treated with nocodazole were much sensitive to cell death, by a factor of approximately 2-3-fold, compared with non-synchronized cells following USP7 knockdown (Figure 3b). Therefore, USP7-depleted mitotic cells undergo apoptosis more effectively than with USP7-depleted non-synchronized cells, indicating that USP7 is important for mitotic progression. 

Then, to examine the presence of alterations in mitosis when USP7 is non-functional, immunostaining was performed to detect the spindle pole protein pericentrin. In control cells, two spindle poles were shown at the proper orientation for cell division (Figure 3c). However, USP7 knockdown induced aberrant spindle poles formation, with both the number and location of poles being altered. In USP7-depleted HeLa cells, we observed the formation of many more than two spindle poles, and they were not properly oriented across from each other (Figure 3c). We quantified the number of mitotic-defective cells, defined by the presence of more than two spindle poles, in USP7-depleted cells (Figure 3d). The total number of mitotic-defective cells was approximately 12 times higher in USP7-depleted cells when compared with control cells (Figure 3d). In addition, the percentage of cells demonstrating chromatin condensation and fragmentation, indicating apoptosis was approximately 15% in USP7-depleted cells vs. less than 2% in control cells (Figure 3e). When caspase 3 activity was measured by fluorogenic substrate in USP7-depleted cells, the relative caspase 3 activity was 7.7 times higher than that of the control cells (Figure 3f). These results show that loss of USP7, which is highly expressed in mitosis, induces multiple spindle pole formation, consequently resulting in mitotic catastrophe. This underlines that USP7 itself is an important factor for mitotic progression. 

### 2.4. Targeting USP7 Increases the Sensitivity of Paclitaxel-Resistant Lung Cancer

Mitotic catastrophe can be induced by treatment with mitotic inhibitors, such as paclitaxel. Taxanes are major chemotherapeutic drugs that target microtubules and induce mitotic catastrophe [21,22]. Long-term treatment with these agents frequently induces chemoresistance, a main obstacle to effective chemotherapy [28]. Given that paclitaxel-resistant cancer has resistance to mitotic catastrophe [29], and depletion of USP7 induces mitotic defects and mitotic catastrophe, we next wanted to investigate whether targeting USP7 induces apoptosis in paclitaxel-resistant cancer. 

**Figure 4:**
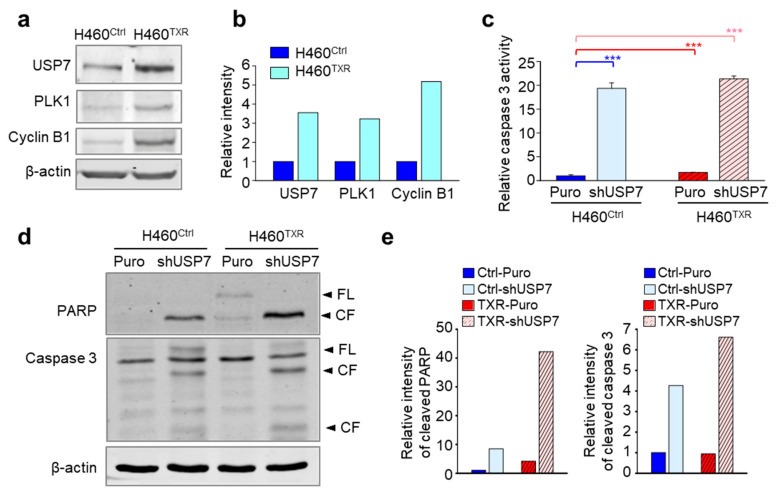
Targeting USP7 increases the sensitivity of paclitaxel-resistant lung cancer. (**a**) Immunoblot analysis was performed to detect the levels of USP7, PLK1, and cyclin B1 in NCI-H460 and NCI-H460^TXR^ cells using specific anti-USP7, anti-PLK1, anti-cyclin B1, and anti-β-actin antibodies. (**b**) The band intensities from the immunoblots in Figure 4a were quantified USP7, PLK1, and cyclin B1 relative to the intensities of β-actin were quantified using LI-COR Odyssey software (Li-COR Biosciences), normalized, and plotted. (**c**–**e**) USP7 was depleted in NCI-H460 and NCI-H460^TXR^ cells using lentiviral USP7 shRNA. (**c**) Cell lysates from NCI-H460 and NCI-H460^TXR^ cells were subjected to a fluorometric caspase 3 activity assay. The means ± SDs (error bars) of data from at least three experiments are shown. (*** *p* ≤ 0.001) (**d**) Immunoblot analysis was done to detect the levels of cleaved PARP and cleaved caspase 3 after USP7 depletion in NCI-H460 and NCI-H460^TXR^ cells. (**e**) The band intensities of cleaved PARP (left) and cleaved caspase 3 (right) from the immunoblots in Figure 4d relative to the intensities of β-actin were quantified using LI-COR Odyssey software (Li-COR Biosciences), normalized, and plotted.

First, we evaluated the levels of USP7 protein using immunoblotting in parental NCI-H460 and paclitaxel-resistant NCI-H460 (NCI-H460^TXR^) cells (Figure 4a,b). The protein levels of USP7 and the mitotic factors PLK1 and cyclin B1 were higher in NCI-H460^TXR^ cells compared to NCI-H460 cells. Because USP7 levels were higher in paclitaxel-resistant NCI-H460^TXR^ cells compared to non-resistant control cells, USP7 was depleted in NCI-H460 and NCI-H460^TXR^ cells to further investigate this up-regulation. Then, caspase 3 activity was measured to determine whether USP7 depletion induces apoptosis more sensitively in NCI-H460^TXR^ cells compared to non-resistant parental cells (Figure 4c). Figure 4c shows that caspase 3 activity was much higher in NCI-H460^TXR^ cells than in parental NCI-H460 cells following USP7 depletion. To confirm these apoptotic patterns in USP7-depleted NCI-H460^TXR^ and NCI-H460 cells, immunoblot analyses were performed using anti-PARP and anti-caspase 3 antibodies (Figure 4d). The levels of cleaved PARP and cleaved caspase 3 were approximately 4 times and 1.5 times higher, respectively, in USP7-depleted NCI-H460^TXR^ cells compared to USP7-depleted parental NCI-H460 cells (Figure 4e). These results demonstrate that USP7 depletion induces apoptosis more effectively in NCI-H460^TXR^ cells than parental NCI-H460 cells, indicating that USP7 inhibition may improve the sensitivity of taxane-resistant cancer to apoptosis.

### 2.5. Combination Treatment with the USP7 Inhibitor P22077 and PLK1 Inhibitor Volasertib Shows Synergic Anticancer Effects in Paclitaxel-Resistant Lung Cancer

**Figure 5:**
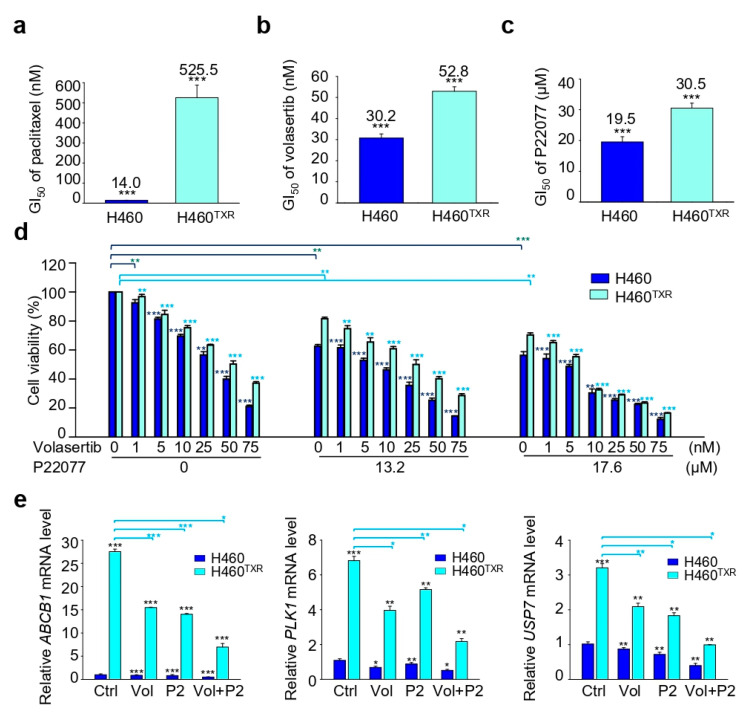
Combination of the USP7 inhibitor P22077 and PLK1 inhibitor volasertib shows synergic anticancer effects in paclitaxel-resistant lung cancer. (**a**–**c**) NCI-H460 and NCI-H460^TXR^ cells were treated with paclitaxel (**a**), volasertib (**b**), or P22077 (**c**) for 48 h in a concentration-dependent manner. The percentages of viable cells were measured by a cell viability assay. The bar graph presents the mean values of half maximal growth inhibitory concentration (GI_50_). (**d**) Combination treatment with volasertib and P22077 was performed in NCI-H460 and NCI-H460^TXR^ cells. Cells were grown for 48 h in the presence of 0, 13.2, or 17.6 µM P22077 and volasertib at the indicated concentrations. The percentages of viable cells were measured by a cell viability assay. (**e**) QRT-PCR was performed for *ABCB1*, *PLK1*, and *USP7* in NCI-H460 and NCI-H460^TXR^ cells treated with 14 nM volasertib (Vol) and 17.6 µM P22077 (P2) for 48 h. Three independent experiments were performed and comparisons within groups were presented as the mean ± SDs. (* *p* ≤ 0.05; ** *p* ≤ 0.01; *** *p* ≤ 0.001).

Previous studies have revealed that PLK1 is highly expressed in several malignancies, and that PLK1 is involved in chemoresistance [30,31]. Moreover, PLK1 is highly expressed in paclitaxel-resistant prostate and lung cancer [30]. To investigate the possibility of improving the efficacy of current treatment regimens for paclitaxel-resistant lung cancer, a paclitaxel-resistant lung cancer cell line was co-treated with the PLK1 inhibitor volasertib combined with the USP7 inhibitor P22077 (Figure 5). First, a cell viability assay was performed to investigate cell viability following treatment with paclitaxel, volasertib, or P22077 alone. The GI_50_ of paclitaxel was 37.5 times higher in paclitaxel-resistant lung cancer NCI-H460^TXR^ than in parental NCI-H460 cells, demonstrating that the resistance index (RI) was 37.5 (Figure 5a). The GI_50_ of the PLK1 inhibitor volasertib and USP7 inhibitor P22077 were measured following treatment with volasertib or P22077 in NCI-H460^TXR^ and NCI-H460 cells (Figure 5b,c). When cells were treated with volasertib or P22077 alone, the GI_50_ values of both volasertib and P22077 were approximately 1.5-fold higher in NCI-H460^TXR^ cells compared to parental NCI-H460 cells, indicating that volasertib and P22077 are equally effective in inhibiting the growth of paclitaxel-resistant NCI-H460^TXR^ cells and parental NCI-H460 cells.

To understand their efficacy for arresting cancer cell growth, the cells were co-treated with the PLK1 inhibitor volasertib and USP7 inhibitor P22077 (Figure 5d, Table 1). The concentration of P22077 was fixed at 13.2 µM or 17.6 µM, which corresponds to the GI_20_ and GI_30_ values in NCI-H460^TXR^ cells, respectively, while volasertib was dosed in a concentration-dependent manner in both NCI-H460 and NCI-H460^TXR^ cells (Figure 5d). The combination index (CI) value was measured following combination treatment with volasertib and P22077. When cells were treated with P22077 at a concentration of 13.2 µM or 17.6 µM, the GI_50_ of volasertib was reduced to 6.4 nM and 2.4 nM, respectively, in NCI-H460 parental cells; by contrast, the GI_50_ of volasertib alone was 30.2 nM.

The CI values were 0.889 and 0.982 in NCI-H460 cells, indicating that combination treatment with volasertib and P22077 showed a synergy in NCI-H460 parental cells (Table 1). Next, we investigated CI values in the paclitaxel-resistant NCI-H460^TXR^ cells. The GI_50_ of volasertib alone was 52.8 nM in NCI-H460^TXR^ cells. However, when NCI-H460^TXR^ cells were co-treated with P22077 at a concentration of 13.2 µM or 17.6 µM, the GI_50_ of volasertib was reduced to 22.9 nM and 5.8 nM, respectively, from 52.8 nM. The CI values were 0.866 and 0.687, respectively, in NCI-H460^TXR^ cells. Therefore, combination treatment with volasertib and P22077 continued to show synergistic activity in paclitaxel-resistant lung cancer.

Then, we investigated whether inhibition of PLK1 and USP7 can regulate the expression of *ABCB1* in NCI-H460^TXR^ cells, because chemoresistance is related with the overexpression of *ABC* transporter and paclitaxel is a substrate of p-glycoprotein encoded by *MDR/ABCB1* [32]. For this, we observed the levels of *ABCB1* by treatment with volasertib and P22077 at the concentrations of 14 nM and 17.6 µM, which corresponds to the GI_30_ values in NCI-H460^TXR^ cells, respectively. The single treatment of volasertib or P22077 reduced the levels of *MDR/ABCB1* in NCI-H460^TXR^ cells (Figure 5e). Combination treatment with volasertib and P22077 markedly reduced the levels of *MDR/ABCB1* in NCI-H460^TXR^ cells. The mRNA levels of *PLK1* and *USP7* were also down-regulated by co-treatment of volasertib and P22077 in NCI-H460^TXR^ cells (Figure 5e). Thus, combination treatment with volasertib and P22077 showed a strong synergism in paclitaxel-resistant lung cancer by down-regulation of *MDR/ABCB1*.

## 3. Discussion

Our previous study revealed that USP7 binds to the 53BP1/PLK1/Aurora A complex to stabilize 53BP1 during mitosis [2]. In addition, we observed that defects in USP7 induced mitotic aberration due to the destabilization of mitotic factors, such as 53BP1 [2]. Additional studies support that USP7 is important in mitotic progression through the stabilization of mitotic PLK1 [3], Aurora A [5], and Bub3 expression [4]. These observations lead us to hypothesize that targeting USP7 would be valuable for cancer treatment, especially in carcinomas that have resistance to mitotic catastrophe. In this study, we clearly demonstrate several important findings. First, USP7 is highly expressed in several carcinoma patients and its expression peaks in mitosis. Second, due to its high expression in mitosis, depletion of USP7 induces apoptosis through mitotic catastrophe in prostate and lung cancer. Third, because targeting USP7 effectively results in mitotic catastrophe in cancer cells, a USP7 inhibitor can exert anticancer activity by inducing apoptosis in carcinomas with resistance to mitotic catastrophe. Fourth, we found that combination treatment with inhibitors of USP7 and the mitotic kinase PLK1 shows a strong synergism in taxane-resistant lung cancer through down-regulation of *MDR1/ABCB1*. 

USP7 plays an important role in the p53-Mdm2 axis by regulating the ubiquitin-proteasome pathway. It removes ubiquitin from ubiquitin-tagged substrate proteins, including Mdm2, thereby protecting against proteasomal degradation. It has been reported that USP7 has a dual function in the tumor suppressor p53-Mdm2 pathway, since it regulates the stability of p53 controversially through the deubiquitination of both p53 and Mdm2 depending on the cellular context [11,16,33,34]. Although USP7 can interact with both Mdm2 and p53 depending on the cellular context, USP7 preferentially forms a stable USP7-Mdm2 complex even in the presence of excess p53 [35], indicating that USP7 predominantly functions to stabilize Mdm2. Thus, this preferential deubiquitination of Mdm2 by USP7 secondarily leads to the degradation of p53, which is one of reasons for carcinogenesis and tumor progression lacking proper cell growth regulation. Loss of USP7 activity enhanced the auto-ubiquitination of Mdm2, resulting in the degradation of Mdm2 and the stabilization of p53 in vivo [36,37,38]. Consistent with these studies, we found that loss of USP7 up-regulates the levels of p53, which triggers p53-dependent apoptosis through Bax expression for apoptotic regulation in NSCLC. Therefore, targeting USP7 would be valuable for inducing apoptotic cell death of NSCLC. Taxane resistance could affect to the levels of Mdm2 and p53 through USP7 up-regulation. Further studies of Mdm2 and p53 by USP7 up-regulation in taxane-resistant cancer would be needed.

The taxanes paclitaxel and docetaxel are widely used as chemotherapeutic agents for cancer treatment, including prostate [24], lung [23,39], and breast cancer [25]. They act by stabilizing microtubules, an essential component of mitotic spindles, thereby inhibiting tubulin depolymerization, which disrupts mitotic spindle formation during mitosis [22]. According to the clinical reports and current protocols for NSCLC treatment [40,41,42], paclitaxel is a valuable chemotherapeutic agent, combined with platinum-based agents such as carboplatin or cisplatin. Even to increase its stability determined by pharmacokinetic parameters in patients, albumin-bound paclitaxel (nab-paclitaxel) had been developed and successfully used in NSCLC [40]. In the clinic, the active use of taxanes or platinum-based agents can induce chemoresistance in patients [28]. Therefore, combination therapy has been recently investigated to overcome this barrier to effective treatment. Previously, we found that PLK1 is highly expressed in taxane-resistant prostate and lung cancer [30]. In addition, we observed that PLK1 inhibition is effective in suppressing the growth of taxane-resistant prostate cancer cells by causing mitotic aberration [30]. Thus, we identified that PLK1 inhibition is a valuable strategy to overcome the limitations of taxane resistance, both alone and in combination with inhibitors of USP7 and PLK1, which are up-regulated in mitosis. These experimental results suggest that targeting mitotic oncogenic factors, such as PLK1 and USP7, may be effective for treating carcinomas that have resistance to mitotic catastrophe. Additional further investigation targeting PLK1 and USP7 would be interested in platinum-resistant lung cancer for clinical usage since platinum-based agents are also first in line for the treatment of NSCLC [40,41,42].

Taxane resistance is related with overexpression of ABC transporters since paclitaxel or docetaxel is a substrate of p-glycoprotein encoded by the *MDR1/ABCB1* [32]. After cloning *ABCB1* in 1985 [43], p-glycoprotein inhibitors have been studied for over four decades. However, until now it is not successful for targeting ABC transporter to remove the chemoresistance. Most trials were terminated due to severe side effects, leaving sparse agents still under consideration for ongoing clinical evaluation. No authority-approved inhibitor of ABC transporters exists until today [44]. Because of current difficulties, we investigated other targets to reduce the chemoresistance. As results of this effort, we revealed that combination treatment with a USP7 inhibitor and PLK1 inhibitor exerts a strong synergistic effect through down-regulation of *MDR1/ABCB1* in taxane-resistant NSCLC cells. Single treatment of inhibitor of USP7 or PLK1 and their combination markedly reduced the expression of *MDR1/ABCB1* in taxane-resistant NSCLC cells. In this view, the inhibitors of USP7 or PLK1 can be effective to reduce the chemoresistance through down-regulation of *MDR1/ABCB1*. 

In summary, here we report that the expression of USP7 is increased in mitosis and targeting USP7 using either a specific inhibitor or shRNA is effective in arresting cellular growth and inducing apoptosis via mitotic aberration in several cancer cell lines. Our results demonstrate that inhibiting USP7 is effective in treating cancer cell lines in vitro, even those that have resistance to mitotic catastrophe. Further in vivo experiments must be done to conclude the combination effects of PLK1 inhibitor and USP7 inhibitor for the clinical application. However, combination treatment with a USP7 inhibitor and PLK1 inhibitor exerts a strong synergistic effect in lung cancer cells resistant to paclitaxel, suggesting that co-targeting USP7 and PLK1 may be a valuable strategy for future clinical translation research. 

## 4. Materials and Methods

### 4.1. Materials

Dulbecco’s modified Eagle’s medium (DMEM), Minimum essential medium (MEM), Roswell Park Memorial Institute (RPMI)-1640 medium, fetal bovine serum (FBS), penicillin, and streptomycin were purchased from Corning Cellgro (Manassas, VA, USA). Volasertib and P22077 were from Selleckchem (Houston, TX, USA) and Lifesensors (Malvern, PA, USA), respectively. All other chemical reagents were from Sigma-Aldrich (St. Louis, MO, USA).

### 4.2. Cell Culture and Establishment of Paclitaxel-Resistant Cancer Cells

Human prostate cancer LNCaP and human lung cancer NCI-H460 and A549 cells were cultured at 37 °C in a 5% CO_2_ humidified atmosphere in RPMI-1640 medium, supplemented with 10% (*v*/*v*) heat-inactivated FBS, 100 units/mL penicillin, and 100 µg/mL streptomycin. Human prostate cancer DU145, cervical cancer HeLa, and non-transformed hTERT-RPE-1 cells were cultured in MEM, DMEM, and DMEM/F12, respectively, supplemented with 10% (*v*/*v*) heat-inactivated FBS, 100 units/mL penicillin, and 100 µg/mL streptomycin. To develop paclitaxel resistance, NCI-H460 cells were exposed to stepwise escalating levels of paclitaxel. The concentration of paclitaxel increased 2-fold at each step of resistance, from 1 nM up to 20 nM. The resistant cells were considered established after 30 weeks of paclitaxel treatment in the NCI-H460^TXR^ cells.

### 4.3. Bioinformatics Analysis

Gene expression profiles were obtained from an online database (https://software.broadinstitute.org/morpheus).

### 4.4. Lentivirus-Based shRNA Preparation and Selection

Lentiviral shRNA transfer plasmids targeting human USP7 (gene access number: NM_ 001286457) at base pair positions 2891 to 2911 (GGACATAGACAAAGAGAATGA) (pLKO-Puro.1-USP7) were prepared, and lentivirus was generated as described previously [2,40]. Infections were carried out in the presence of 10 mg/mL polybrene and 10 mM HEPES. Cells were infected with lentiviral vectors expressing shRNA targeting USP7 or control virus (Control) carrying the pLKO-puro.1 empty vector for 1 day. Samples were prepared after selection with puromycin for 2 days.

### 4.5. Cell Viability Assay

Cell viability was measured using MTT (3-(4, 5-dimethylthiazolyl-2)-2, 5-diphenyltetrazolium bromide) (Sigma) according to the manufacturer’s protocol.

### 4.6. Fluorometric Caspase 3 Activity Assay

Cell lysates (50 µg) were incubated with 200 nM Ac-DEVD-AMC (BD Biosciences, USA) in reaction buffer (20 mM HEPES, pH 7.5, 2 mM DTT, and 10% glycerol) at 37 °C. Per the manufacturer’s protocol, the reaction was monitored by fluorescence emission at 430 nm (excitation at 380 nm).

### 4.7. Western Blotting 

For immunoblotting, cell extracts were prepared by lysing cells in lysis buffer (10 mM HEPES [pH 7.4], 10 mM KCl, 2 mM MgCl_2_, 5 mM EGTA, 25 µg/mL leupeptin, 5 µg/mL pepstatin A, 1 mM phenyl methyl sulfonyl fluoride [PMSF], 40 mM β-glycerophosphate, 1 mM dithiothreitol [DTT]). Cell lysates were centrifuged at 12,000 rpm for 15 min at 4 °C, and the supernatants were collected. After determining the protein concentration of each sample, cell lysates were boiled and resolved by 12% SDS-PAGE before undergoing Western blot analysis with the appropriate antibodies. The anti-PLK1 antibodies were purchased from Upstate (New York, NY, USA). The anti-cyclin B1, anti-PARP, anti-cleaved caspase 3, anti-GAPDH, and anti-β-actin antibodies were from obtained from Santa Cruz (Santa Cruz, CA, USA). Immunoblots were visualized with an Odyssey infrared imaging system (LI-COR Biosciences; Lincoln, NE, USA).

### 4.8. Immunofluorescence 

Cells grown on coverslips were fixed with 4% paraformaldehyde and permeabilized with methanol. Cells were washed three times with 0.1% Triton X-100 in PBS, incubated overnight at 4 °C in 0.1% Triton X-100-PBS containing 3% BSA for blocking, and then incubated with anti-α-tubulin (Sigma-Aldrich) and anti-pericentrin (Abcam) antibodies. Next, cells were washed three times with 0.1% Triton X-100 in PBS and incubated with Cy3-conjugated anti-rabbit or anti-mouse secondary antibodies or FITC-conjugated anti-mouse or anti-rabbit secondary antibodies (Jackson Immuno Research Laboratories; West Grove, PA, USA) and 4, 6-diamidine-2-phenylindole (DAPI; Sigma-Aldrich) to stain nuclear DNA. Images were collected and analyzed using the Z series setting of the Applied Precision Deconvolution Microscope and Delta vision software. For measuring mitotic-defective cells, multipolar spindles were quantified greater than two pericentrin foci in each cell according to the previous reports [2,45]. To measure apoptotic cells, apoptotic characteristic nuclear morphology such as DNA condensation were detected [46]. 

### 4.9. Quantitative Reverse Transcription Polymerase Chain Reaction (qRT-PCR)

Total RNA was extracted 48 h after exposure to volasertib and P22077 and quantified by Nanodrop (Thermo Scientific; Wilmington, DE, USA). cDNA was generated with a First Strand cDNA Synthesis Kit (Thermo Scientific). After the synthesized cDNA was mixed with SYBR Green Master Mix (Bio-Rad; Hercules, CA, USA) and various sets of gene-specific primers, qRT-PCR was performed using a CFX96 Real-Time PCR system (Bio-Rad). Primers used for amplification are 5′-ATATCAGCAGCCCACATCAT-3′ and 5′-GAAGCACTGGGATGTCCGGT-3′ for human ABCB1, 5′-ATTCCTAACATTGCCACCAG-3′ and 5′-TTTACACCATTTGCCATCC-3′ for human USP7, 5′ AAGAGATCCCGGAGGTCCTA-3′ and 5′-TCATTCAGGAAAAGGTTGCC-3′ for human PLK1, 5′ TAAAGGGCATCCTGGGCTACACT-3′, and 5′-TTACTCCTTGGAGGCCATGTAGG-3′ for human GAPDH. Error bars represent the mean ± SD. The significance of differences between the experimental groups was calculated using the *t*-test.

### 4.10. Statistical Analysis

All data are given as means ± SDs. Results were analyzed for statistically significant differences using Student’s *t*-test, and statistical significance was set at *p* < 0.05. (* *p* ≤ 0.05; ** *p* ≤ 0.01; *** *p* ≤ 0.001).

## Figures and Tables

**Table 1:** The half maximal growth inhibitory concentration (GI_50_) values for P22077 and volasertib and their combination index (CI) in H460 and H460TXR cells. CI < 1 represents synergism, CI = 1 represents additive effect, CI > 1 represents antagonism.

	H460^Ctrl^		H460^TXR^	
	GI_50_	CI	GI_50_	CI
P22077 (µM)	19.5		30.5	
Volasertib (nM)	30.2		52.8	
Volasertib (in combination 13.2 µM P22077)	6.4	0.889	22.9	0.866
Volasertib (in combination 17.6 µM P22077)	2.4	0.982	5.8	0.687

## Abbreviations

PLK1	Polo-like kinase 1
USP7	Ubiquitin-specific-processing protease 7
TXR	Taxol Resistance
GI	The growth inhibitory concentration
CI	Combination index
RI	Resistance index
